# Engineering a light-controlled F_1_ ATPase using structure-based protein design

**DOI:** 10.7717/peerj.2286

**Published:** 2016-07-28

**Authors:** Daniel Hoersch

**Affiliations:** Experimental Molecular Biophysics, Department of Physics, Freie Universität Berlin, Berlin, Germany

**Keywords:** Azobenzene, Protein design, Light control, Molecular machine

## Abstract

The F_1_ sub-complex of ATP synthase is a biological nanomotor that converts the free energy of ATP hydrolysis into mechanical work with an astonishing efficiency of up to 100% (Kinosita et al., 2000). To probe the principal mechanics of the machine, I re-engineered the active site of *E.coli* F_1_ ATPase with a structure-based protein design approach: by incorporation of a site-specific, photoswitchable crosslinker, whose end-to-end distance can be modulated by illumination with light of two different wavelengths, a dynamic constraint was imposed on the inter-atomic distances of the α and β subunits. Crosslinking reduced the ATP hydrolysis activity of four designs tested in vitro and in one case created a synthetic ATPase whose activity can be reversibly modulated by subsequent illumination with near UV and blue light. The work is a first step into the direction of the long-term goal to design nanoscaled machines based on biological parts that can be precisely controlled by light.

## Introduction

ATP-driven protein machines are fundamental to life. They perform extraordinarily complex and diverse biological functions: DNA replication/transcription (helicases, DNA/RNA polymerases), intracellular trafficking (myosin, kinesin, dynein), ATP production (ATP synthase), protein folding/unfolding (chaperonins, HSP90, proteasome) or maintenance of ion gradients (V-ATPase) just to name a few. However, despite the considerable amount of work invested in characterizing the static and dynamic structural features of these big protein complexes, the understanding of their detailed molecular mechanism has been limited in large by the complexity of the allosteric coupling, which converts the chemical energy of ATP hydrolysis into large-scale conformational changes.

**Figure 1:**
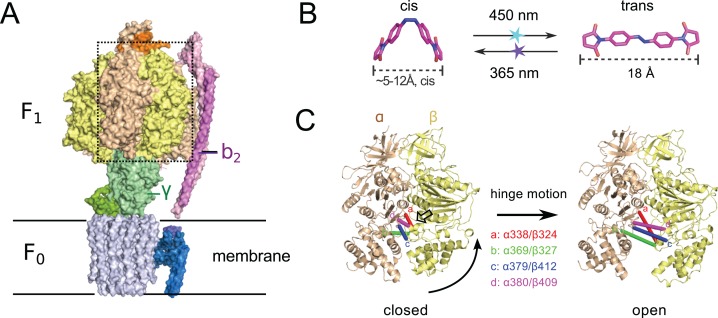
(A) Crystal structure of ATP synthase from *Paracoccus denitrificans* (Morales-Rios et al., 2015). (B) Chemical structure of the crosslinker ABDM in the two isomerization states *cis* and *trans* (models created with the software Avogadro (Hanwell et al., 2012)). (C) Conformational re-arrangements of the α/β dimer that switches between the closed and open conformation of the nucleotide binding cleft. Colored bars connect the C_α_ atoms of the sequence positions that are promising targets for site-specific crosslinking with ABDM: αS338/βQ324 (a, red), αN369/βS327 (b, green), αG379/βG412 (c, blue), αA380/βV409 (d, magenta). A transparent arrow indicates the location of the active site.

To tackle this problem, predictive engineering is a promising way to rigorously test and improve mechanistic models of complex systems. An example of such an approach is the successful reprogramming of the homo-oligomeric group II chaperonin Mm-cpn to use light instead of ATP hydrolysis to open and close around an internal cavity by artificially constraining its conformational space (Hoersch & Kortemme, 2016; Hoersch et al., 2013). This was realized this by site-specific crosslinking of neighbouring subunits of the protein complex with the thiol-reactive molecular spacer azobenzene-dimaleimide (ABDM, Fig. 1B), that reversibly switches inter-atomic distances upon illumination with two different wavelengths of light, due to the reversible *trans-cis* photoisomerization of the azobenzene group. Controlling the activity of biological and bioactive molecules with the high spatial and temporal resolution of light has a rich history, dating back to the 1970s with the introduction of caged compounds (Beharry & Woolley, 2011; Kaplan, Forbush & Hoffman, 1978; Mayer & Heckel, 2006; Szymański et al., 2013). The crosslinking of proteins with azobenzene bearing compounds has been harnessed previously to control the secondary structure of peptides and proteins (Kumita et al., 2003; Kumita, Smart & Woolley, 2000; Zhang et al., 2010), to modulate the accessibility of a ligand to ion channels and receptors (Banghart et al., 2004; Numano et al., 2009; Volgraf et al., 2006), to modulate the activity of an enzyme (Schierling et al., 2010), to control the dimerization properties of catherin (Ritterson et al., 2013), and to regulate an ATP-driven protein translocation system (Bonardi et al., 2010).

Another interesting target system for predictive re-engineering using azobenzene based crosslinker is F_0_F_1_ ATP synthase, a complex, physiologically important and highly efficient biological machine (Junge & Nelson, 2015; Okuno, Iino & Noji, 2011; Walker, 2013). The protein complex consists of two fully reversible rotary motor units F_0_ and F_1_, which are coupled by a rotor (γ) and a stator unit (b_2_) (Fig. 1A). Dependent on the conditions the system either synthesizes ATP using a trans-membrane proton gradient or pumps protons when hydrolyzing ATP. In both cases the energy is transmitted between the F_0_ and F_1_ units via the torque of the rotating γ unit. Under ATP synthesis conditions this rotation drives the active site, which is located at the interface of the α/β subunits of the F_1_ unit (Fig. 1A) to undergo a conformational cycle that supplies the energy necessary for ATP synthesis. This structural change mainly consists of a hinge-bending motion of the β-subunit that opens up the active site for product release (Fig. 1C). F_0_F_1_ can be disassembled in vitro resulting in a F_1_ sub-complex that rotates the γ subunit upon ATP hydrolysis (Duncan et al., 1995; Noji et al., 1997; Sabbert, Engelbrecht & Junge, 1996) and synthesizes ATP if the γ unit is rotated using an external force (torque) (Itoh et al., 2004; Rondelez et al., 2005).

To dissect the mechanical coupling of the ATP binding pocket of F_1_ ATPase it might be interesting to apply an engineering strategy similar to the one applied to Mm-cpn: By picking crosslinking sites in-between the α and β subunits of *E.coli* F_1_, that are supposed to undergo a distance change during the hinge bending motion that matches the distance change of ABDM during the *cis*→*trans* photoisomerization (Fig. 1B), it should be possible to perturb the motor function of F_1_ by artificially constraining the mobility/flexibility of the machinery of the motor in a light-dependent fashion.

Here I report a first successful step into this direction: The design and manufacturing of four double cysteine mutants of *E.coli* F_1_ ATPase for ABDM crosslinking. Incubation with ABDM leads to a formation of covalently linked α/β dimers within the F_1_ complex and a significant decrease of the ATP hydrolysis activity for all tested mutants. In the case of the ABDM coupled mutant αA380C/βV409C, the ATP hydrolysis activity can furthermore be modulated by subsequent illumination with blue and near UV light. This behavior can be explained with the different distance constraints the two isomers of ABDM impose on the geometry of the active site.

## Materials and Methods

### 

#### Structure-based design

To design attachment sites for ABDM, the crystal structure of *E.coli* F_1_, ATPase with a resolution of 3.26 Å was used (PDB ID: 3OAA). The expected distances between sulphur atoms for every possible pair of cysteines mutations in neighbouring α/β units (harbouring the active site) as well as the expected solvent accessible surface area of the sulphur were calculated using the software PyMOL (Schroedinger, LCC). The data set was then screened for residue pairs with an expected sulphur distance of 5–14 Å in the closed and 16.5–20.5 Å in the open state and a minimum expected solvent accessible surface area for the sulphur atoms of 20 Å^2^ (25% of the maximum solvent accessible surface area of the sulphur atom in a deprotonated cysteine residue), leading to 39 residue pairs satisfying the matching criteria. This list was visually inspected for candidates with a large distance change between the closed and open state and for which there is enough unoccupied space in between the attachment sites to accommodate ABDM. Four candidate sequence positions for crosslinking were chosen for experimental testing: α338/β324, α369/β327, α379/β412 and α380/β409.

#### Plasmids

pKH4 a plasmid containing the operon of a functional *E.coli* F_0_F_1_ ATPase synthase in which all native cysteines are replaced with alanines and a 6xHis-tag is attached to the N-terminus of the β subunit was a gift from W. Junge and S. Engelbrecht (Kuo, Ketchum & Nakamoto, 1998; Noji et al., 1999). All cysteine double mutants were produced by site-directed mutagenesis with the Quickchange method (Aligent Genomics). The sequences of the primers used for site-directed mutagenesis are listed in the Supplemental Material. The incorporation of the cysteine mutations was confirmed by plasmid sequencing (Microsynth AG, Balgach, Switzerland).

#### Protein purification

The *E.coli* F_1_ ATPase double cysteine mutants were expressed in *E.coli* BL21-CodonPlus-RP cells (Aligent Technologies) grown in LB medium and purified via affinity chromatography using a Ni-NTA agarose resin (Macherey-Nagel, Düren, Germany) as described previously (Greene & Frasch, 2003). The presence of the α, β and γ subunits in the purified samples indicates the correct folding and assembly of the F_1_ complex.

#### ABDM crosslinking and photoswitching

ABDM was purchased from BIOZOL (BIOZOL Diagnostica Vertrieb GmbH, Eching, Germany). ABDM was dissolved in DMF to a concentration of 1.2 mM and stored at −20 °C. ABDM was added to a 0.3 mg/ml solution of the F_1_ mutants in buffer A (20 mM HEPES pH 7.4, 100 mM KCl, 5 mM MgCl_2_, 5% glycerol) at a ratio of 1 μl ABDM solution per 50 μl protein solution. The crosslinking reaction was incubated for at least 2 h at room temperature and then quenched by addition of 1 mM DTT. To shift the azobenzene isomer equilibrium, samples were illuminated for 10 s with either a blue 3 W LED (447 nm, LUXEON Rebel) to accumulate the *trans* state or a 3 W UV LED (365 nm, LED Engin) to accumulate the *cis* state. Longer illumination times did not change the *trans*/*cis* isomer equilibrium (Fig. S2). The crosslinking ratio of the samples was determined via analysis on a 8% sodium dodecyl sulphate (SDS)-PAGE gel by calculating the intensities of the α/β dimer band relative to the α and β monomer bands with the ImageJ software package (Schneider, Rasband & Eliceiri, 2012).

#### UV-VIS spectroscopy

The absorption spectra shown in Figs. S1 and S2 were recorded with a Shimadzu UV-2450 UV-VIS spectrometer. The samples were prepared by washing and concentrating ABDM crosslinked F_1_ αA380C/βV409C in buffer A using an Amicon-ultra 0.5 ml centrifugal filter with 50 kDa pore size (Millipore, Billerica, MA, USA).

#### ATPase assay

F_1_ samples were diluted 1:100 in buffer A to a concentration of ∼7 nM and incubated with 0.1 mM ATP for 20 min at room temperature (For the photoswitching experiment shown in Fig. 3 the protein was diluted 1:50). The increase in concentration of inorganic phosphate (P_i_) in the sample was assayed using a Malachite Green Phosphate Assay Kit (Cayman Chemical Company, Ann Arbor, MI, USA). The absorption at 620 nm, indicative of the presence of the green molybdophodphoric acid complex was measured against buffer with a UV-VIS spectrometer (IMPLEN Nanophotometer, München, Germany). The P_i_ concentration of the sample was calculated from the 620 nm absorption of the sample using a calibration curve measured with buffer A supplemented with P_i_ at known concentrations. Under the tested conditions the ATP hydrolysis activity of the double cysteine F_1_ mutants was at least 50% of the parent cysteine-less F_1_ construct.

## Results and Discussion

### Structure-based design of F_1_ double cysteine mutants

In a computational screen of the crystal structure of the asymmetric *E.coli* F_1_ ATPase unit (PDB ID: 3OAA (Cingolani & Duncan, 2011)) four promising crosslinking sites in between the α and β subunits were identified, whose distances for the closed and open ATP binding cleft match the end-to-end distance of ABDM in the *cis* and *trans* isomerization states (see Fig. 1B and Table 1). Cysteines for crosslinking were introduced by site-directed mutagenesis into a plasmid with a functional cysteine-free ATP synthase operon.

**Table 1:** Results of a computational screen for ABDM crosslinking sites. Res α and Res β are the amino acid sequence positions chosen for cysteine mutation in the α and β subunit of F_1_. Dist () refers to the expected distance of the sulphur atoms of the engineered cysteines in neighbouring subunits (see Fig. 1B). SASA_SG_ () is the expected surface accessible area for the deprotonated SG atom of the corresponding cysteine. “cl” and “op” denote the open and closed ATP binding pocket, respectively.

PDB ID	Res α	Res β	Dist (cl) (Å)	SASA_SG_ (α, cl) (Å^2^)	SASA_SG_ (β, cl) (Å^2^)	Dist (op) (Å)	SASA_SG_ (α, op) (Å^2^)	SASA_SG_ (β, op) (Å^2^)
3OAA	338	324	7.40	63	75	19.73	71	65
	369	327	8.66	27	43	20.41	27	67
	379	412	6.11	43	81	19.87	69	57
	380	409	5.82	64	64	16.96	75	41

### ABDM crosslinks the double cysteine mutants with varying efficiency

**Figure 2:**
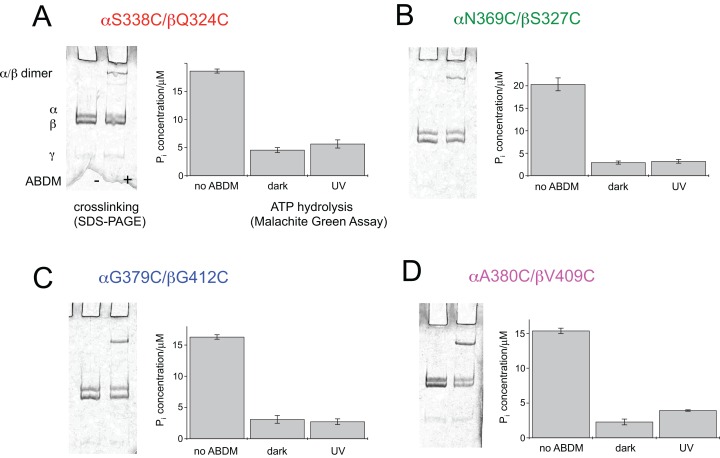
ABDM crosslinking and ATPase activity of the F_1_ double cysteine mutants. (A) αS338C/βQ324C, (B) αN369C/βS327C, (C) αG379C/βG412C and (D) αA380C/βV409C. (A, C) Depict SDS-PAGE gels of the mutants before and after incubation with ABDM. The appearance of a shifted high molecular weight band indicates the formation of crosslinks between the α and β subunit. (B, D) Show the F_1_ catalyzed increase of the P_i_ concentration of inorganic phosphate (P_i_) after ATP incubation of the sample for the F_1_ double cysteine mutant (no ABDM), the *trans*-ABDM crosslinked F_1_ mutant (dark) and the ABDM crosslinked F_1_ mutant after UV light illumination (UV). Shown are the mean values and standard error of three independent experiments.

After expression and purification the F_1_ units the double cysteine mutants were incubated with ABDM in the *trans* isomerization state (*trans*-ABDM). The formation of covalently crosslinked α/β dimers was monitored on a SDS PAGE-gel (Fig. 2). From the intensities of the monomer and dimer bands it is possible to estimate the crosslinking ratio of the samples defined as the number crosslinks divided by the number of possible crosslinking sites (Hoersch et al., 2013). The crosslinking ratios for the different mutants are 0.2 for ABDM-αS338C/βQ324C and ABDM-αN369C/βS327C, 0.3 for ABDM-αG379C/βG412C and 0.4 for ABDM-αA380C/βV409C. These numbers are below one, the value for full crosslinking; note however that the α_3_/β_3_ barrel of the F_1_ sub-complex is asymmetric in the presence of the γ unit and only one of the three active sites is in the open conformation (Abrahams et al., 1994; Cingolani & Duncan, 2011). This limits the maximal achievable crosslinking ratio.

### ABDM crosslinking reduces the ATP hydrolysis activity of F_1_

For the same set of samples the ATPase activity was determined using the colorimetric Malachite Green assay. The assay senses the presence of inorganic phosphate in the sample after incubation with ATP via complex formation with malachite green molybdate. The complex absorbs at 620 nm and its concentration can be determined using a conventional UV-VIS spectrometer. Figure 2 shows that for all double cysteine mutants ABDM-crosslinking leads to a decrease of the ATP hydrolysis activity of the sample. The ATPase activity decreases for the different ABDM-incubated mutants to a fraction between 0.14 and 0.24. This behavior is in line with the design strategy as the ABDM crosslinker is supposed to constrain the flexibility of the active site by stabilizing either the open (*trans*-ABDM) or closed (*cis*-ABDM) conformation of the nucleotide binding pocket. Note that the ATPase activity of F_1_ is highly cooperative therefore controlling the conformation of one αβ dimer might be sufficient to control the ATPase activity of the whole F_1_ complex (Boyer, 1997; Milgrom & Cross, 2005).

### The ATPase activity of ABDM-αA380C/βV409C is light dependent

**Figure 3:**
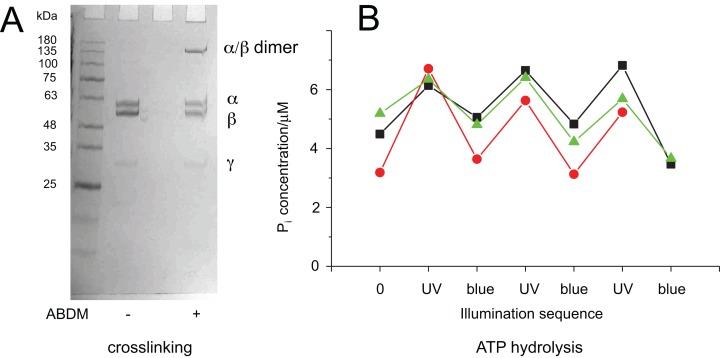
Light-dependent ATPase activity of ABDM-crosslinked F_1_ αA380C/βV409C. (A) SDS-PAGE gels of the mutant before and after incubation with ABDM. The appearance of a shifted high molecular weight band indicates the formation of crosslinks between the α and β subunit (crosslinking ratio: 0.3). (B) F_1_ catalyzed increase of the P_i_ concentration after ATP incubation for a *trans*-ABDM crosslinked F_1_ mutant sample after subsequent illumination with UV and blue light. The data refer to three experiments involving independent crosslinking, illumination and ATPase activity assay. The sample shown in the right lane of the SDS-PAGE gel in panel A refers to the red trace (spheres).

To test if the ATP hydrolysis activity depends on the isomerization state of the crosslinker the Malachite Green assay was further performed after illumination of the ABDM crosslinked F_1_ sub-complexes with light at 365 nm, which induces a *trans*→*cis* photoisomerization of the azobenzene group of ABDM. For 3 of the 4 crosslinked F_1_ samples (ABDM-αS338C/βQ324C, ABDM-αN369C/βS327C and ABDM-αG379C/βG412C) UV light illumination did not result in significant change of the ATP hydrolysis activity (Figs. 2A–2C). For ABDM-αA380C/βV409C however, UV light induced an increase in ATP hydrolysis activity (Fig. 2D). To test if this light-dependent change in ATPase activity is reversible, the same assay was performed on three independently crosslinked αA380C/βV409C samples that were exposed to alternating illumination at 365 and 450 nm (Note that 450 nm illumination induces a *cis*→*trans* photoisomerization ABDM). Figure 3 shows that for all 3 samples illumination leads to a reversible modulation of the ATP hydrolysis activity. UV light induced *trans*→*cis* isomerization of ABDM increases the activity and blue light induced *cis*→*trans* isomerization decreases the activity over several illumination cycles. UV-VIS absorption spectroscopy confirms fully reversible *trans/cis* photoswitching of ABDM in ABDM-αA380C/βV409C under the applied illumination conditions (Fig. S1) (Umeki et al., 2004).

It is possible to explain this behavior with the different molecular properties of the two isomerization states of ABDM: *trans*-azobenzene is predicted to be more rigid than *cis-*azobenzene (Fig. 1B). For the calculated distance distributions of the azobenzene isomerization states, see Beharry et al., 2012; Schafer et al., 2007; Zhang et al., 2009. In a consequence *trans*-ABDM likely imposes a stronger distance constraint on the conformation of the active site of F_1_ than *cis*-ABDM. As the active site of F_1_ has to switch constantly between the open and closed conformation for ATP binding, hydrolysis and product release, *trans*-ABDM might therefore perturb the conformational cycle more efficiently than *cis*-ABDM. This behavior is exactly what is observed in the experiment.

Previously crosslinking via engineered disulfide bridges has been used to constrain the rotation of the γ unit in respect to the α3/β3 barrel of the F_1_ unit and to determine interaction between subunits of the F_0_ unit of ATP synthase (DeLeon-Rangel et al., 2013; Duncan et al., 1995; Sielaff et al., 2008). However, to my knowledge the strategy of crosslinking with azobenzene bearing compounds has not been applied to F_0_F_1_ ATP synthase so far.

## Conclusions

This work shows for the first time that it is possible to manipulate the molecular machinery of *E.coli* F_1_ ATPase reversibly in a light-dependent fashion using the photoswitchable molecular spacer ABDM. It is an initial step in the direction of re-engineering protein-based molecular motors using the azobenzene-based spacer to constrain and manipulate the position of moving parts of the machinery. In future experiments I want to test if the azobenzene spacer is able to actively drive the conformational cycle of the active site of F_1_ ATPase using the energy of the photo-isomerizing azobenzene. This will help to elucidate the design principles and dynamic properties of biological motors and might in the long run inspire the bottom-up design of synthetic nano-scaled machines based on biological parts.

## Supplemental Information

10.7717/peerj.2286/supp-1 Supplemental Information 1 Supplemental Data and Figures.

10.7717/peerj.2286/supp-2 Supplemental Information 2 Data for Fig. 2A.

10.7717/peerj.2286/supp-3 Supplemental Information 3 Data for Fig. 2B.

10.7717/peerj.2286/supp-4 Supplemental Information 4 Data for Fig. 2C.

10.7717/peerj.2286/supp-5 Supplemental Information 5 Data for Fig. 2D.

## Additional Information and Declarations

### Data Deposition

The following information was supplied regarding data availability: The raw data has been supplied as Supplemental Dataset Files.
