# Supplementary material for: Engineering a light-controlled F1 ATPase using structure-based protein design
Source: PeerJ. 2016 Jul 28;4:e2286. doi: 10.7717/peerj.2286 (PMC4974930; doi:10.7717/peerj.2286)
Supplement: Supplemental Information 1 [file peerj-04-2286-s001.pdf]

Supplemental Figures:

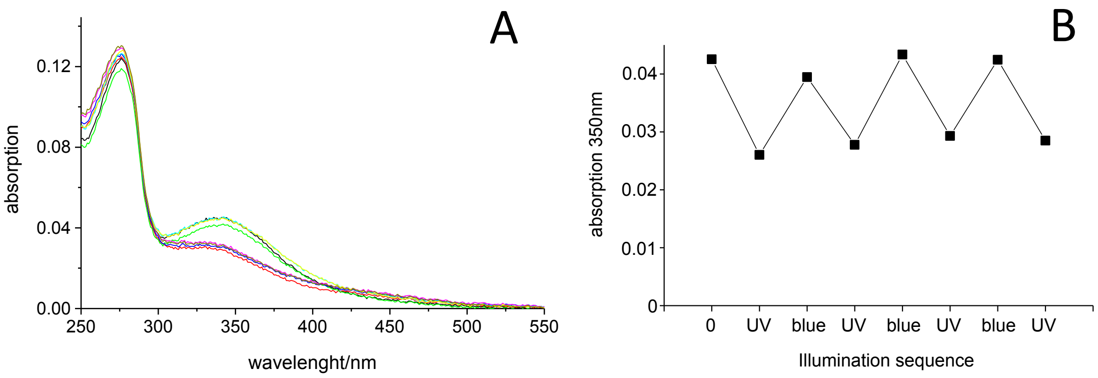

Figure S1: Consecutive UV and blue light illumination reversibly switches the trans/cis isomer equilibrium of ABDM bound to the F_1 double cysteine mutant αA380C/βV409C **A** absorption spectra of ABDM-αA380C/βV409C before illumination (black) and after sequential illumination at 365nm (red) 450nm (green) 365nm (blue) 450nm (cyan) 365nm (magenta) 450nm (yellow) and 356nm light (dark yellow). **B** 350nm absorption of the samples shown at panel A.

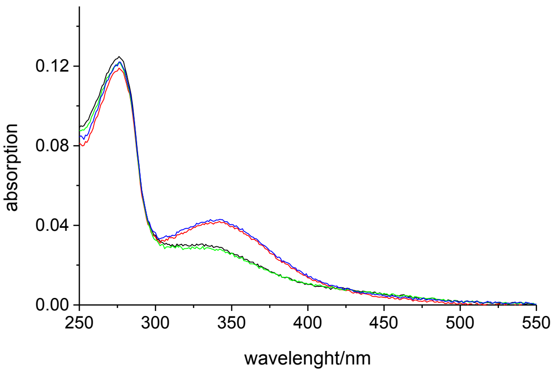

Figure S2: Additional illumination with 365nm and 450nm does not change the cis/trans isomer equilibrium of ABDM conjugated F_1 αA380C/βV409C. Absorption spectra of ABDM-αA380C/βV409C after sequential illumination with 3W LEDs for 10sec at 365nm (dark) for another 10 seconds at 365nm (green) for 10sec at 450nm (red) and another 10sec at 450nm (green).

Supplemental Data:

Primer for site-directed Mutagenesis:

αS338C
forward: caggcgggtgacgtttGtgcgttcgttccgacc
reverse: ggtcggaacgaacgcaCaaacgtcacccgcctg

βQ324C
forward: gtggtactgagccgtTGCatcgcgtctctgggt
reverse: acccagagacgcgatGCAacggctcagtaccac

αN369C
forward: attcgtcctgcggttTGcccgggtatttccgta
reverse: tacggaaatacccgggCAaaccgcaggacgaat

βS327C
forward: agccgtcagatcgcgtGtctgggtatctacccg
reverse: cgggtagatacccagaCacgcgatctgacggct

αG379C
forward: gtatcccgtgttggtTgtgcagcacagaccaag
reverse: cttggtctgtgctgcacAaccaacacgggatac

βG412C
forward: gcagaagtattcaccTgttctccgggtaaatac
reverse: gtatttacccggagaacAggtgaatacttctgc

αA380C
forward: tcccgtgttggtggttgcgcacagaccaagatc
reverse: gatcttggtctgtgcgcaaccaccaacacggga

βV409C
forward: ttcttcgtggcagaatgcttcaccggttctccg
reverse: cggagaaccggtgaagcattctgccacgaagaa
